# Pathological femoral neck fracture caused by an echinococcus cyst of the vastus lateralis - case report

**DOI:** 10.1186/1471-2334-11-103

**Published:** 2011-04-21

**Authors:** János Csotye, Krisztián Sisák, Loránt Bardócz, Kálmán Tóth

**Affiliations:** 1 Department of Traumatology, Békés County Hospital, 1 Semmelweis Street, Gyula, 5700, Hungary; 2 Department of Orthopaedics, University of Szeged, 6 Semmelweis Street, Szeged, 6720, Hungary

**Keywords:** echinococcus, cyst, femur, fracture, prosthesis

## Abstract

**Background:**

Musculoskeletal hydatid cysts are rare, but being locally invasive, can potentially cause significant deformity or pathological fracture.

**Case presentation:**

A 39 y.o. male presented to our orthopaedic outpatient clinic complaining of severe right hip pain, and inability to ambulate. Symptoms were not preceded by trauma. Subsequent imaging confirmed a large, 17 × 3 × 5 cm echinococcus cyst in the vastus lateralis, causing erosion of the proximal metaphysis of the femur. As a consequence the patient suffered a non-traumatic pathological intertrochanteric femur fracture. The patient was treated with an en-bloc excision of the lesion - the affected soft tissue envelope containing the large cyst - and as a second surgical step a cemented total hip replacement (THR) was implanted under the same anaesthetic.

The manuscript reviews the literature regarding musculoskeletal hydatid disease.

## Background

Among Echinococcus species, E. granulosus and E. multilocularis are commonly reported to be responsible for human hydatidosis. The endemicity of this parasitic infection may vary across countries. Echinococcus larvae develop in cystic form, mostly in the liver or the lungs. The involvement of other organs, and particularly the muscles, is relatively rare. Only 0.5 to 2% of hydatid cysts are skeletally localized [1].

In this paper we report a rare case of a hydatid cyst located in the soft tissues of the right thigh and involving the proximal femur.

### Case presentation

A 39-year-old male was referred by his family physician to our out-patient clinic, complaining of right-sided thigh pain. He had recently moved to our region from Romania (a hyperendemeic area for echinococcosis) and worked as a shepherd.

His previous history included a routine right sided inguinal hernia repair, because of persistent groin pain 9 years previously. As there was no symptomatic improvement after his hernia repair anterior-posterior and lateral radiographs of the affected hip were obtained, showing a cyst in the femoral neck. A few months after the hernia repair, the cyst was explored, curreted and the cavity was bone grafted using corticospongiosus autograft from the iliac crest. The histological examination from the excised tissue showed the presence of Echinococcus granulosus.

**Figure 1:**
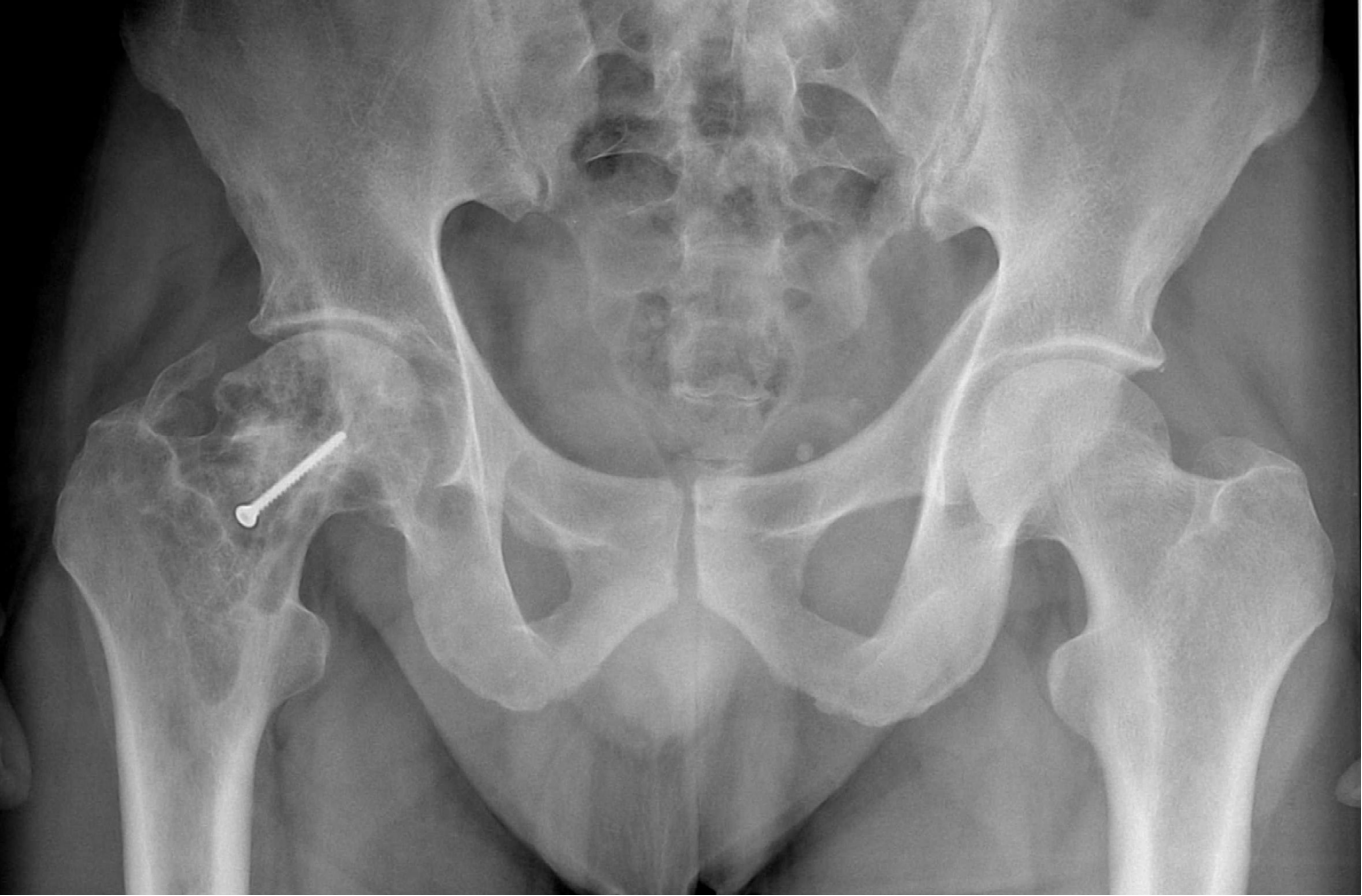
**AP Pelvis radiograph showing a multicystic lesion in the right proximal femur with a cortical screw in situ**.

The radiographs taken at his recent presentation, 8 years after the previous surgery, showed a multiloculated cyst around a previously implanted cortical screw, which was fixing the bonegraft (Figure 1).

**Figure 2:**
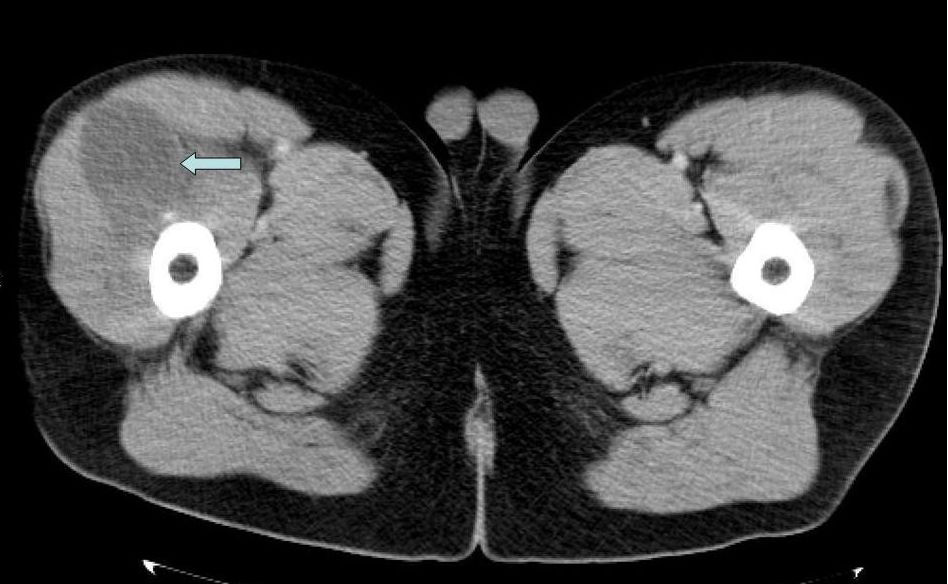
**A coronal slice of the CT scan performed showing a large soft tissue mass on the anterolateral part of the right thigh**.

We were aware of the previous histological results which were positive for *E. granulosus*. Blood tests showed a high serological titer for *E. granulosus*, but otherwise normal values. To investigate for other potential parasitic sites a CT scan of the brain, skull, chest, abdomen and pelvis was performed, with special attention to the right thigh. The scan confirmed a large (17 × 3 × 5 cm) cyst in the anterolateral part of the proximal thigh within the vastus lateralis (Figure 2). The CT scan is essential in determining the extent of bony disease, especially in the proximal femur. If hip replacement is necessary, the feasibility of using routine implants depends on the destruction of proximal femoral structures, best shown by a CT. If the calcar and/or the greater trochanter have to sacrificed, the surgical plan (implant use, rehabilitation, potential outcome) changes. There were no other cysts visible.

**Figure 3:**
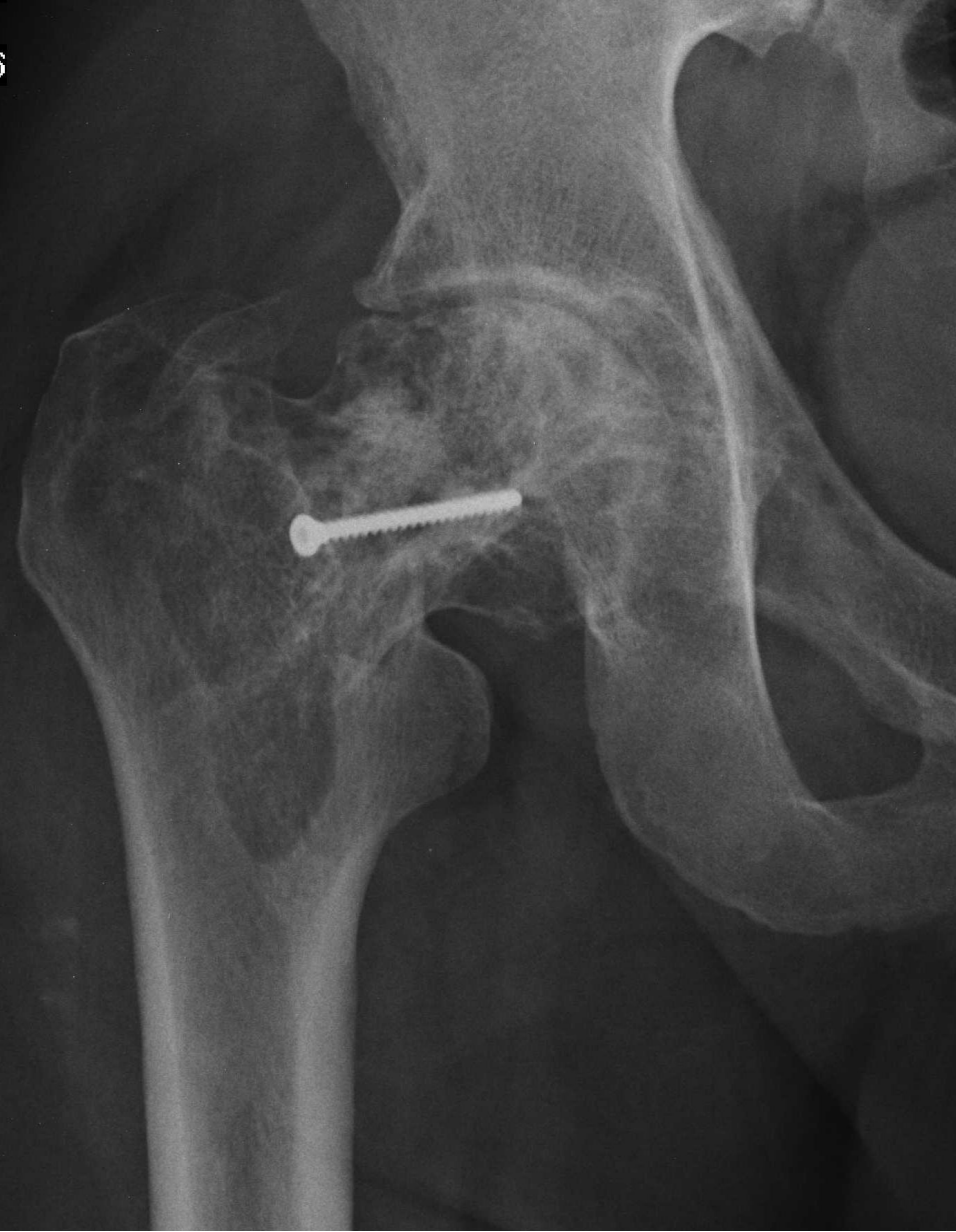
**AP radiograph of the right hip showing a basicervical femoral fracture**.

The patient was scheduled for a relatively urgent elective operation, because of the prefracture state and was discharged. Two days prior to the planned procedure, he represented with severe thigh pain, and inability of weight-bearing on the affected side. Subsequent imaging confirmed a non-traumatic pathological fracture in the intertrochanteric region (Figure 3).

A two step procedure was performed under the same anesthetic, utilizing an extended lateral (Hardinge) approach, with distal extension. During the first part of the procedure an en-bloc excision of the cyst was performed. The lesion was localized between the vastus lateralis and rectus femoris muscles. It had eroded the anterior cortex of the proximal metaphysic, just above the lesser trochanter. After thorough debridement, an adjuvant was used, which is well-established in tumor surgery. The bony bed was exposed to a concentrated phenol solution (70%) via a soaked gauze, followed by copious lavage with a concentrated alcohol solution. No spillage or anaphylaxis was observed during the first part of the procedure.

During the second stage of the procedure, a cemented THR was implanted using an Aesculap implant (Centrament stem, PE-CUP, 28 mm isodur head). The patient received oral Mebendazole (Vermox, Richter Gedeon), 400 mg bid, preoperatively for 5 days. Unlike with intraabdominal pathology, preoperative chemotherapy was only possible for a short period of time, as emergent fracture treatment was necessary. The chemotherapy was continued for 6 weeks postoperatively using the same agent and dose (Mebendazole). The role and length of post-operative adjuvant chemical therapy in musculoskeletal hydatid disease is unclear. In alveolar echinococcosis, it is recommended that chemotherapy is continued for up to 2 years after surgery. The role of life-long chemosuppression is being explored [2].

**Figure 4:**
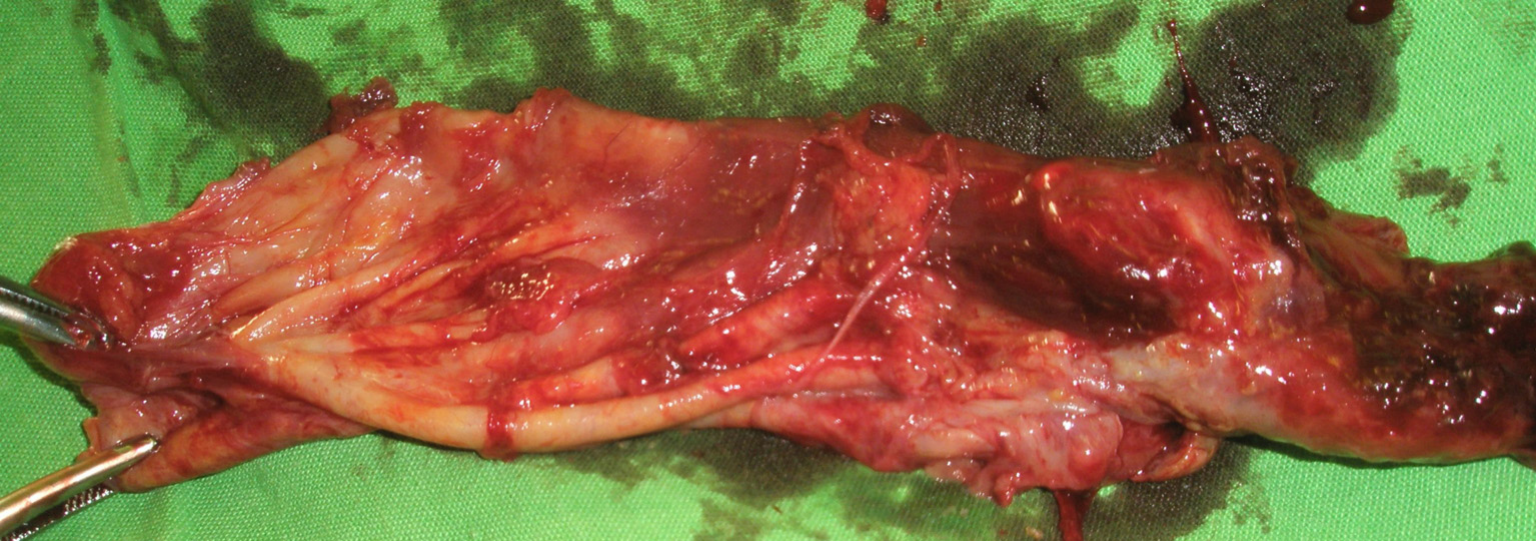
**Intraoperative photo of the excised soft tissue lesion after en-bloc surgical excision**.

**Figure 5:**
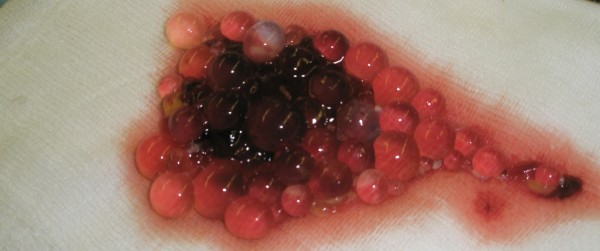
**Contents of the cyst showing small, grape-sized vesicles**.

**Figure 6:**
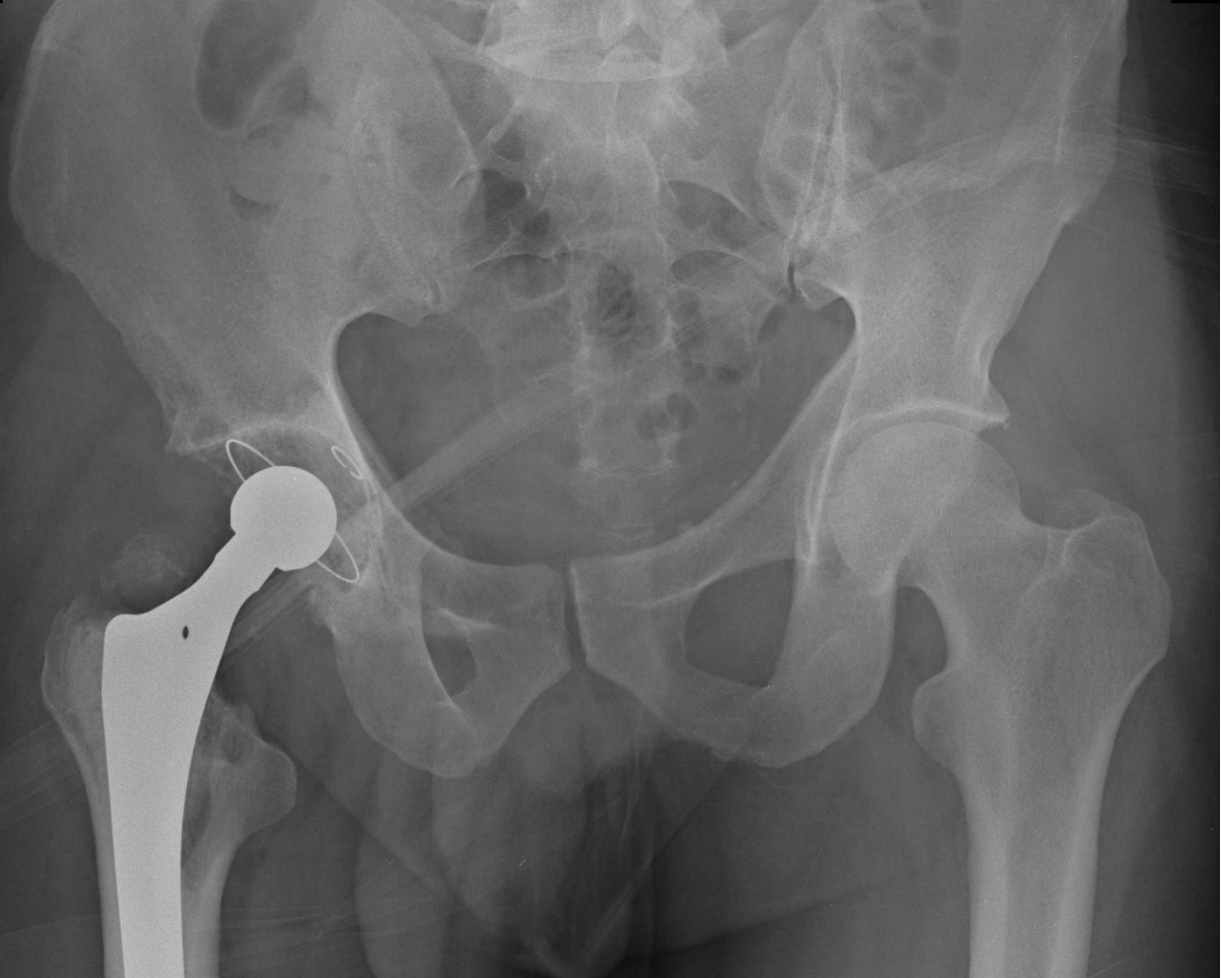
**1 year postoperatively, the AP pelvic radiograph shows a well placed THR with no loosening**.

Several samples were taken intraoperatively. The histopathological examination confirmed the suspected diagnosis (Figure 4 and 5). The patient had an uneventful postoperative period, with uncomplicated wound healing. He was asymptomatic at the latest follow-up, one year postoperatively, with a normal plain radiograph (Figure 6). Inflammatory markers are within the normal threshold, however the serological titer remains high (>1024). Patient remains under close (3 monthly) follow-up.

## Conclusion

Echinococcosis (or hydatid disease) is a parasitic disease usually infecting mammals (mainly sheep, dogs, rodents and horses). The disease has a worldwide geographical distribution, with clear evidence of the emergence of the condition in parts of eastern Europe [3]. In its adult stage, the parasite resides in the bowels of the animal. The larvae of *E. granulosus *infect humans, as an intermediate host, giving rise to cystic lesions, which are most frequently found in the liver and the lungs, but very rarely in bone (0.9-2.5%) [4]. The *E. granulosus *larvae develop via exogenous vesiculation in bones, due to the mechanical stress present. This can eventually lead to mechanical weakening and destruction of the bone trabeculae. The lesions are infiltrative in nature and progress slowly, with the development of numerous microvesicles, but without the parasite encysting. The clinical signs are nonspecific and location-dependent.

In many respects, the disease is reminiscent of chronic osteomyelitis, fibrous dysplasia, osteosarcoma, benign cystic bone lesions, lesions caused by hyperparathyroid tumors and other neoplastic lesions [5]. Due to its prognosis it has been referred to as ''white cancer'' [6]. Standard radiographs usually reveal poorly defined lytic lesions in a classical honeycomb pattern. There is periosteal reaction with localized decalcification [7]. The soft tissue and visceral locations can be explored for abscesses by ultrasonography [7], whilst CT allows a complete workup of the most lesions. Differential diagnosis can be difficult. An early diagnosis is essential if the affected bone is to be fully eradicated and the healthy tissue salvaged.

In case of bony involvement, the disease has a high recurrence rate of 50%. It however, almost never recurs if the primary lesion is in muscle - 0% [8]. If muscles or bones are affected the most commonly used therapeutic regimen is radical surgical excision combined with adjuvant pre- and postoperative chemical therapy. Mebendazole has been the agent of choice historically, for adjuvant chemotherapy. Although no data exists on chemotherapy in musculoskeletal echinococcosis, in patients having intraabdominal pathology, albendazole, especially in combination with praziquantel has proven to be more effective in achieving high serum and cyst fluid levels of albendazole sulphoxide [9].

Locally aggressive, benign bone tumors require specific techniques to avoid recurrence, thus the experience from their treatment can be used as an analogy for the treatment of musculoskeletal hydatidosis. Surgeons usually perform intralesional curettage, together with some form of adjuvant therapy (the use of intralesional curettage alone can be followed by a recurrence rate of up to 50%). The most commonly applied adjuvant therapies/procedures are cautery, high-speed burring, exothermic cement polymerization and the use of certain solutions (e.g. phenol or, more rarely, alcohol) [10,11]. Capanna et al. reported on a series of 165 benign bone tumors with a high rate of recurrence (e.g. aneurysmal bone cysts, chondroblastomas and giant cell tumors) and noted that the utilization of phenol decreased the local recurrence rate from 41% to 7% [12].

In our specific case, wide, radical surgical excision of the cyst would have necessitated removal most of the proximal metaphysis, and hence the implantation of a tumorprosthesis.

In view of the patient's age and active life style, we opted to perform an en-bloc excision with a relatively small safe zone, thus sparing both trochanters (with the abductors) and the calcar. To reduce the likelihood of recurrence, 70% phenol solution was used as an adjuvant. The exothermic reaction of cementing can also contribute in preventing recurrence. Using a more limited excision, perhaps carries a higher risk of recurrence. In a young and active patient if the disease recurs, a tumoprosthesis remains a surgical option as a revision procedure. A THR provides a significantly better quality of life in the short term.

The necessary long and extensive surgery carries a higher risk of infection and sepsis, with a substantial risk of death [13]. The use of antibiotic laden antibiotic cement is advisable and might reduce infection risk, therefore we use a pre-mixed dual-combination antibiotic-loaded bone cement (Copal G+C, G = gentamicin, C = clindamycin; Heraeus Medical GmbH, Germany).

In the current English language literature, there are only two cases describing the removal of a primary echinococcus cyst which was eventually followed by successful hip replacement several years later [14,15]. Replacing the affected hip carries a very high risk for subsequent complications and failure of the implant [16,17]. Due to the high potential failure rate the patient was informed in detail regarding potential complications and recurrence.

We were unable to find a case report describing a primary echinococcus cyst causing a pathological femoral neck fracture where a single operation was successfully used to treat the cyst and to implant a THR.

## Abbreviations

THR: Total Hip Replacement.

## Competing interests

The authors declare that they have no competing interests.

## Authors' contributions

All authors contributed to this work. JC, LB, KS, KT were involved in the direct clinical care (diagnosis, decision making, treatment, operative intervention, postoperative care) of the patient and/or the preparation of this manuscript. All authors read and approved the final manuscript.

## Pre-publication history

The pre-publication history for this paper can be accessed here:

http://www.biomedcentral.com/1471-2334/11/103/prepub

## References

[B1] Zlitni M Ezzaouia K Lebib H Karray M Kooli M Mestiri M Hydatid Cyst of Bone: Diagnosis and Treatment World J Surg 2001 25 75 82 10.1007/s002680020010 11213159

[B2] McManus DP Zhang W Li J Bartley PB Echinococcosus Lancet 2003 362 1295 304 10.1016/S0140-6736(03)14573-4 14575976

[B3] Eckert J Schantz P Gasser R Eckert J, Gemmell MA, Meslin F-X, Pawlowski ZS Geographic distribution and prevalence WHOI/OIE manual on echinococcosis in humans and animals: a public health problem of global concern 2001 aris: World Organisation for Animal Health 100 41

[B4] Bel Hadj Youssef D Loussaief C Ben Rhomdhane F Chakroun M Abid A Bouzouaia N Kyste hydatique primitif intraosseux: à propos de deux cas Rev Med Int 2007 28 4 255 258 10.1016/j.revmed.2006.12.011 17335941

[B5] Siwach R Singh R Kadian VK Singh Z Jain M Madan H Singh S Extensive hydatidosis of the femur and pelvis with pathological fracture: a case report Int J Infect Dis 2009 13 6 e480 482 10.1016/j.ijid.2008.12.017 19342261

[B6] Chiboub H Boutayeb F Wahbi S El Yacoubi M Ouazzani N Hermas M Echinococcose osseuse du basin Rev Chir Orthop 2001 87 397 401 11431637

[B7] Zlitni M Kooli M Ezzaouia K Lebib H Mestiri M Manifestations osseuses des parasitoses Encycl Med Chir (Elsevier, Paris), Appareil locomoteur 1996 14 021-B10

[B8] Arazi M Erikoglu M Odev K Memik R Ozdemir M Primary echinococcus infestation of the bone and muscles Clin Orthop Relat Res 2005 432 234 241 15738827 10.1097/01.blo.0000149816.86222.2d

[B9] Cobo F Yarnoz Sesma B Fraile P Aizcorbe M Trujillo R Diaz-de-Liano A Ciga MA Albendazole plus praziquantel versus albendazole alone as pre-operative treatment in intra-abdominal hydatidosis caused by Echinococcus granulosus Tropical Medicine and International Health 1998 3 6 462 466 10.1046/j.1365-3156.1998.00257.x 9657508

[B10] Gitelis S Mallin BA Piasecki P Turner F Intralesional excision compared with en bloc resection for giant-cell tumors of bone J Bone Joint Surg Am 1993 75 1648 1655 8245057 10.2106/00004623-199311000-00009

[B11] O'Donnell RJ Springfield DS Motwani HK Ready JE Gebhardt MC Mankin HJ Recurrence of giant-cell tumors of the long bones after curettage and packing with cement J Bone Joint Surg Am 1994 76 1827 1833 7989388 10.2106/00004623-199412000-00009

[B12] Capanna R Sudanese A Baldini N Campanacci M Phenol as an adjuvant in the control of local recurrence of benign neoplasms of bone treated by curettage Ital J Orthop Traumatol 1985 11 381 388 4086284

[B13] Sapkas GS Stathakopoulos DP Babis GC Tsarouchas JK Hydatid cyst of the bones and joints. 8 cases followed for 4-16 years Acta Orthop Scand 1998 69 89 94 10.3109/17453679809002364 9524526

[B14] Kural C Ugras AA Sungur I Ozturk H Erturk AH Unsaldi T Hydatid bone disease of the femur Orthopedics 2008 31 712 19292369

[B15] Moretti B Panella A Moretti L Garofalo R Notarnicola A Giant primary muscular hydatid cyst with a secondary bone localization Int J Infect Dis 2009 in press 10.1016/j.ijid.2009.07.003 19889561

[B16] Notarnicola A Panella A Moretti L Solarino G Moretti B Hip joint hydatidosis after prosthesis replacement Int J Infect Dis 2010 in press 10.1016/j.ijid.2009.12.006 20400352

[B17] Neelapala VS Chandrasekar CR Grimer RJ Revision hip replacement for recurrent Hydatid disease of the pelvis: a case report and review of the literature J Orthop Surg Res 2010 5 17 10.1186/1749-799X-5-17 20222941 PMC2847990

